# Effects of Inactivated *Bordetella pertussis* on Phosphodiesterase in the Lung of Ovalbumin Sensitized and Challenged Rats

**DOI:** 10.1155/2014/581738

**Published:** 2014-07-09

**Authors:** Ya-Juan Wang, Shun-De Song, Jun-Chun Chen, Xue-Feng Wang, Ya-Li Jiang, Qiang-Min Xie, Ji-Qiang Chen, Zi-Gang Li, Hui-Fang Tang

**Affiliations:** ^1^Zhejiang Respiratory Drugs Research Laboratory of SFDA of China, School of Medicine, Zhejiang University, Hangzhou 310058, China; ^2^Clinical College of Integrated Traditional and Western Medicine, Anhui University of Chinese Medicine, Hefei 230038, China; ^3^First Affiliated Hospital, Zhejiang University, School of Medicine, Hangzhou 310000, China; ^4^Second Affiliated Hospital, Zhejiang Chinese Medical University, Hangzhou 310005, China; ^5^Women's Hospital, School of Medicine, Zhejiang University, Hangzhou 310006, China

## Abstract

This paper indicated that inactivated *Bordetella pertussis* (iBp) can enhance the lung airway hyperreactivity of the rats sensitized and challenged with OVA. The mechanisms were involved in the upregulation of cAMP-PDE activity and PDE4A, PDE4D, and PDE3 gene expression in the lungs. But only PDE4 activity was different between the OVA and OVA+iBp groups, and PDE4D expression was significantly increased in iBp rats alone. So, our data suggested that cosensitization with OVA and iBp affects lung airway reactivity by modulating the lung cAMP-PDE activity and PDE4D gene expression.

## 1. Introduction 

Inactivated* Bordetella pertussis* (iBp) has been used as a strong Th2 adjuvant to boost allergic responses to antigen such as house dust mite antigen (HDM), ovalbumin (OVA), and ragweed pollen in animal models of asthmatic hypersensitivity from 1968 [1–4]. Systemic administration of iBp enhances these sensitization processes and enhances the pulmonary and systemic immune responses to locally administered HDM [5]. Our experiments have also suggested that simultaneous exposure to OVA and intramuscularly iBp can enhance the bronchial hyperresponsiveness [6]. But how this occurs at the molecular level has not been elucidated.

The phosphodiesterase (PDE) superfamily participates in the only cellular pathways for degradation of the ubiquitous intracellular second messengers. It comprises eleven biochemically and pharmacologically distinct enzyme families (PDEs 1-11) that hydrolyze cAMP and/or cGMP [7]. PDE4 is specific for cAMP and comprises four subtypes (A, B, C, and D). It is predominantly expressed and plays an important role in the regulation of cellular functions in inflammatory and immune cells. There has been significant interest in PDE4 inhibitors as a potential therapy for inflammatory diseases such as allergy and asthma [8]. Cyclic adenosine monophosphate (cAMP) relaxes airway smooth muscles in the lung. Our previous study using iBp adjuvants suggested that PDE4 is upregulated in the lung of allergic rats [6]. But whether the adjuvants had effects on PDE activity and expression was unclear.

Growing evidence suggests that the D subtype of PDE4-PDE4D plays a key role in balancing relaxation and contraction in airway smooth muscle [9]. The airway smooth muscle contractility of PDE4D-deficient mice is disrupted and no longer responsive to cholinergic stimulation [10]. Interestingly, animals exposed prenatally but not postnatally to cigarette smoke show increased airway hyperresponsiveness after a single intratracheal injection of* Aspergillus fumigatus* extract. This increased airway hyperresponsiveness is causally related to decreased lung cAMP levels, increased PDE4 enzymatic activity, and PDE4D isoform-specific mRNA expression in the lung [11].

Therefore, we set out to investigate the effects of OVA and iBp in airway responsiveness and the possible role of phosphodiesterase. Our study suggested that the response of the airways was different between OVA model with iBp (OVA+iBp) and OVA model without iBp (OVA). Interestingly, PDE4D expression was also increased in the lung of allergic rats using iBp adjuvants, while this result was not observed in the allergic rats without using iBp adjuvants. So, our study first suggested that PDE4D upregulation was induced by iBp and was involved in airway hyperresponsiveness.

## 2. Materials and Methods 

### 2.1. Animal Model Preparation [12]

Male Sprague-Dawley rats (140–160 g, Laboratory Animal Center of Zhejiang University School of Medicine, Hangzhou, China) were maintained under a 24 h light/dark cycle with food and water* ad libitum*. Animals were treated in accordance with the National Institutes of Health Guide for the Care and Use of Laboratory Animals. Animal experiments were approved by the Zhejiang Medical Laboratory Animal Administration Committee.

Rats were sensitized by subcutaneous injection (1 mL) of a saline suspension containing 0.2% OVA (Sigma, St. Louis, MO) and 10% aluminum hydroxide into two footpads, neck, back, groin (0.1 mL each), and abdomen (0.5 mL) on day 0, with or without intramuscular injection of 2 × 10^10^ heat-killed* B. Pertussis* (iBp; 1 mL) into the hindlimbs. Other rats were sensitized with OVA as above but without iBpinjection. From day 14 after sensitization, those rats were challenged once daily for 7 days by 20 min of exposure to aerosolized 1% OVA in saline generated by a jet nebulizer (PARI MASTER, Pari GmbH, Starnberg, Germany; droplet diameter: 1–5 *μ*m).* B. pertussis* control (iBp) rats received intramuscular injection of 2 × 10^10^ iBp (1 mL) only. Blank control rats were “sensitized” and “challenged” with a saline aerosol.

### 2.2. Measurement of Airway Hyperresponsiveness (AHR) [13]

The rat was tracheal cannulated after anesthetized with urethane (40 mg/kg), then placed in a whole-body plethysmograph for the measurement of lung resistance (*R*_*L*_) and lung dynamic compliance (Cdyn) with a real-time data analysis system (MedLab Biological Signal Collection System V 5.0, Medease Scientific Technic Co. Ltd. Nanjing, China). After 5 min for stabilization, AHR was induced by exposure to methacholine aerosols (MCh, Sigma Chemical Co., St. Louis, MO) of increasing doses (0.2, 0.4, 0.8, 1.2, 1.6, 2.0, 4.0, 8.0, 12, and 16 g/L) through the tracheal cannula for 10 s with the jet nebulizer. The signals from all pressure transducers were continuously processed by fitting flow, volume, and pressure to an equation of motion. *R*_*L*_ and Cdyn were monitored for 5 min and maximal changes from baseline for each parameter were recorded. Ten- to fifteen-minute intervals were allowed between concentrations.

### 2.3. Preparation of Bronchoalveolar Lavage Fluids (BALF)

After the last OVA challenge, rats were anaesthetized with urethane (2 g/kg, i.p.). Bronchoalveolar lavage was performed by gently instilling D-Hanks' solution into the lung* via* a tracheal catheter followed by withdrawal. This process was repeated three times with a total volume of 5 mL D-Hanks. Total BALF cells counts were determined with a hemocytometer. Cell pellets' slides were stained with Wright's stain; the number of neutrophils, eosinophils, lymphocytes, and monocytes on each slide was recorded. The total number of cells in each sample was then determined according to the volume of BALF recovered.

### 2.4. Histological Examination

The lungs were infused via the trachea with 1 mL of 10% neutral formalin and immersed in the same fixative for seven days. Tissues were paraffined, and 5 *μ*m sections were cut and stained with H&E for examining cell infiltration under a light microscope. Inflammatory cell infiltration and peribronchial inflammatory cell counts were used to evaluate the severity.

### 2.5. Assays for cAMP-PDE and cGMP-PDE Activities [14]

On day 21, animals were sacrificed, and the lungs were immediately removed, frozen in liquid nitrogen, and then stored at −80°C until analysis. The frozen lungs were thawed and cut into small cubes. Twenty-five milligrams of the lung was homogenized in 100 *μ*L of ice-cold 30 mM HEPES (pH 7.4) containing 0.1% Triton X-100. The PDE assay mixture (200 *μ*L) in PBS (pH 7.4) contained 137 mM NaCl, 2.7 mM KCl, 8.8 mM Na_2_HPO_4_, 1.5 mM KH_2_PO_4_, 1 mM CaCl_2_, 1 mM MgCl_2_, 1 *μ*M cAMP or 1 *μ*M cGMP (Sigma), and lung homogenate. The PDE reaction was started by the addition of 10 *μ*L lung homogenate and was performed at 37°C for 10 min. The reaction was stopped by boiling the mixture for 3 min. The assay mixture was cooled on ice, followed by centrifugation at 12,850 ×g for 30 *μ*min at 4°C. The amount of cAMP or cGMP present in the supernatant was determined by HPLC (Hypersil ODS 4.0 × 250 mm; Hewlett-Packard, Palo Alto, CA) using a standard curve for cAMP. The PDE inhibitors, theophylline (nonselective), rolipram (PDE4-selective), SKF94836 (PDE3-selective), and zaprinast (PDE5-selective), were used to inhibit the PDE activity and to analyze the components of PDE activity in the lung. In brief, 100 *μ*M PDE inhibitor was added to the PDE assay mixture as above and then mixed with lung homogenate. The rest of the process was as above. The total amounts of protein were determined by the Bradford method using BSA as standard [15]. The results were expressed as nanomoles of PDE activity per milligram of protein.

### 2.6. Analysis of PDE Subtype mRNAs

Total RNA was isolated from each tissue frozen in liquid nitrogen using TRIzol reagent (Invitrogen, Carlsbad, CA). Preparation of first-strand cDNA from rat and total RNA was performed using First-Strand cDNA Synthesis kit (Shanghai Sangon Biological Engineering Technology and Service, Shanghai, China) and Advantage RT-for-PCR kit (Clontech, Palo Alto, CA), respectively. The PCR primer sets of PDE were listed in Table 1. Each of the PCR primer sets was able to detect all known variants derived from the appropriate PDE gene. PCR amplification was performed in a PCR buffer (10 mM Tris-HCl, pH 9.0, 100 mM KCl, 80 mM (NH_4_)_2_SO_4_, and 0.1% NP-40) containing 0.2 mM of each dNTP, 1.5 mM of MgCl_2_, 500 nM of each primer, and 1 U of Taq DNA polymerase (Sangon) in a total volume of 25 *μ*L for 30 cycles, with the following cycle parameters: denaturing, 94°C for 45 sec; annealing, 58°C for 70 sec; and extension, 72°C for 2 min. After PCR amplification, 8 *μ*l of each reaction mixture was resolved by electrophoresis on a 1.5% agarose gel containing ethidium bromide, and the PCR product bands were quantified using the UVP Gel Documentation system (UVP, Upland, CA). The levels of PDE mRNAs were calculated relative to *β*-actin. It was confirmed that under these conditions the PCR product accumulation did not reach plateau levels (data not shown).

### 2.7. Statistical Analysis

Data are expressed as mean ± SEM. Statistical analysis was performed with one-way ANOVA (SPSS11.0, USA) to evaluate PDE activity, PDE mRNA expression and lung resistance, and dynamic compliance. Differences with *P* < 0.05 were considered statistically significant.

## 3. Results

### 3.1. Intramuscular iBp Enhanced the AHR

**Figure 1:**
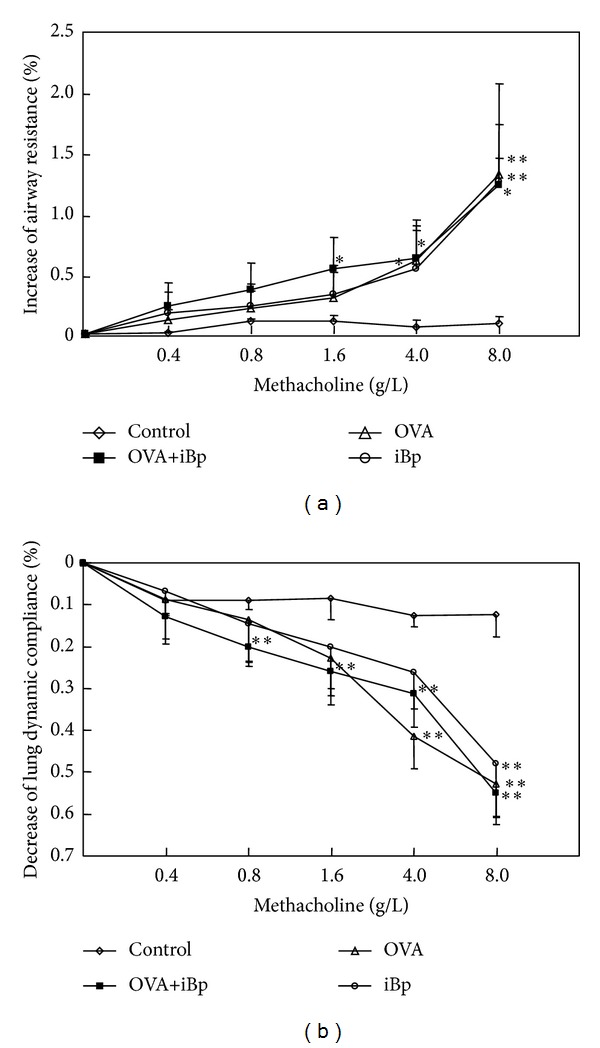
(a) *R*_*L*_ as a marker of AHR induced by MCh in normal (*n* = 6), OVA+iBp (*n* = 8), iBp (*n* = 6), and OVA rats (*n* = 6); (b) Cdyn as a marker of AHR induced by MCh (mean ± SEM; *P < 0.05; **P < 0.01versus normal rats).

The baseline values before aerosol challenge with MCh were similar in control rats, OVA sensitized and challenged rats with iBp as an adjuvant (OVA+iBp), iBp alone (iBp), and OVA sensitized and challenged rats without iBp (OVA). There were no significant differences in basal *R*_*L*_ and Cdyn among groups. Inhaled MCh caused a dose-dependent bronchoconstriction that peaked within 60 s. In our rat model of allergic asthma, OVA sensitization and challenge caused a significant decrease in Cdyn and increase in *R*_*L*_ compared to control rats (Figure 1). Simultaneous exposure to OVA and intramuscular iBp enhanced the decrease in Cdyn and increase in *R*_*L*_ at lower concentrations of MCh, but at high MCh concentrations, the responses of the OVA and OVA+iBP groups were equivalent. Interestingly, a single iBp injection had an effect similar to OVA sensitization and challenge.

### 3.2. Intramuscular iBp Increased the Eosinophils in BALF

To investigate the molecular mechanisms underlying the differences between our rat models, we used another series of rats to study the BALF and the lung. The total number of cells (Table 2) and eosinophils in BALF increased in the OVA+iBp and OVA groups, as compared to controls (*P* < 0.01). At the same time, the numbers of total leukocytes, eosinophils, and neutrophils in BALF from OVA+iBp group were significantly higher than the OVA group. While iBp groupshowed a total number similar to the blank control group, the eosinophils and neutrophils were significantly higher than those in the blank control.

### 3.3. Simultaneous Exposure to Ovalbumin and Intramuscularly iBp Enhanced cAMP-PDE Activity

**Figure 2:**
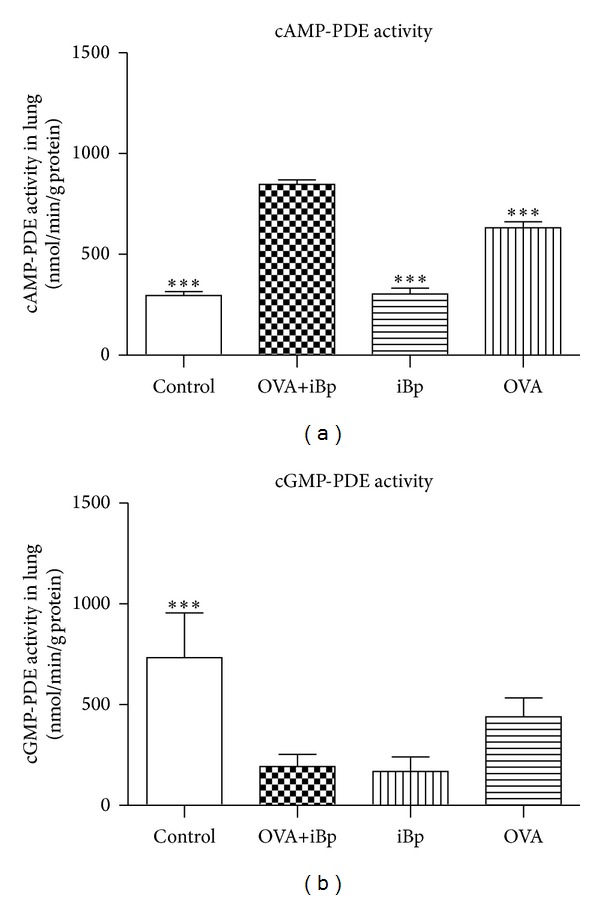
(a) Lung cAMP-PDE activity in normal (*n* = 8), OVA+iBp, iBp (*n* = 6), and OVA rats (*n* = 8); (b) lung cGMP-PDE activity in normal (*n* = 8), OVA+iBp, iBp (*n* = 6), and OVA rats (*n* = 8) (mean ± SEM; *P < 0.05; **P < 0.01 versus OVA+iBp; ^#^ *P* < 0.05; ^##^ *P* < 0.01 versus normal).

With regard to PDE regulation in the lung, OVA sensitization and challenge with or without iBp significantly elevated the cAMP-PDE activity, resulting in 2.5-fold or 2-fold higher activity than that of normal animals, although the difference between OVA and OVA+iBp groups was not statistically significant (Figure 2(a)). But iBp did not affect the cAMP-PDE activity; the level was equivalent to the normal control. The total cGMP-PDE activity in the normal control and the OVA groups was high, while in OVA+iBp and iBp groups the activity was decreased (Figure 2(b)).

**Figure 3:**
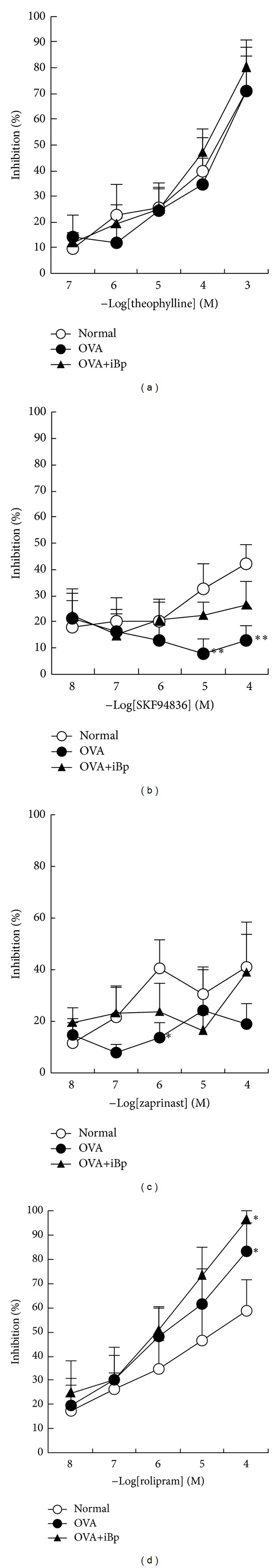
Inhibitory effects (%) of theophylline (a), SKF94836 (b), zaprinast (c), and rolipram (d) on the cAMP-PDE activity in the lung homogenates harvested from OVA+iBp and OVA rats (mean ± SEM; *P < 0.05; **P < 0.01 versus normal).

**Figure 4:**
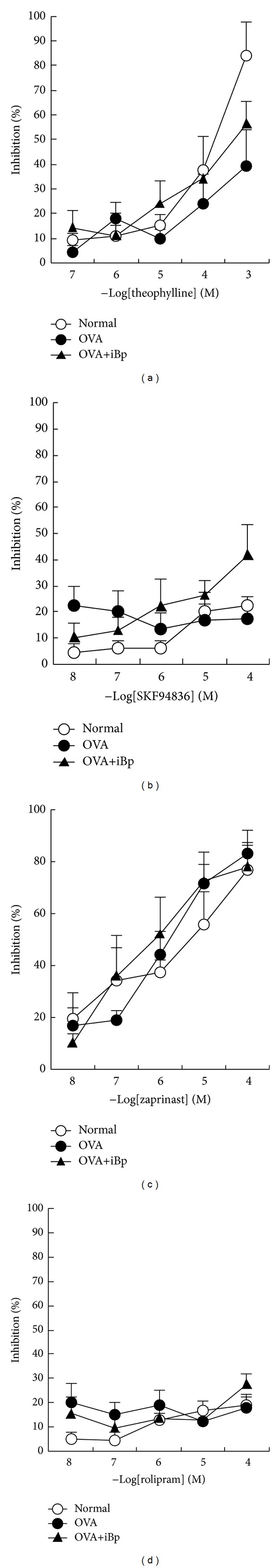
Inhibitory effects (%) of theophylline (a), SKF94836 (b), zaprinast (c), and rolipram (d) on the cGMP-PDE activity in the lung homogenates harvested from OVA+iBp and OVA rats (mean ± SEM).

To further determine which PDE subtype plays a key role in the total PDE activity, we used the theophylline (nonselective PDE inhibitor), rolipram (PDE4 inhibitor), SKF94836 (PDE3 inhibitor), and zaprinast (PDE5 inhibitor) to determine the overall PDE activity of each subtype. Rolipram (100 *μ*M) sensitive cAMP-PDE activity (i.e., PDE4 activity) was 1.4-fold or 1.6-fold higher in the OVA or OVA+iBp rats than in normal rats (Figure 3(d)). These results suggest that the increase in total cAMP-PDE activity by OVA sensitization and challenge is primarily due to upregulation of PDE4. Zaprinast (100 *μ*M) sensitive cGMP-PDE activity (i.e., PDE5 activity) was the major resource of cGMP-PDE activity, but the OVA or the OVA+iBp rats were similar to normal rats (Figure 4(c)).

### 3.4. Intramuscular iBp Enhanced mRNA Expression of PDE4D

**Figure 5:**
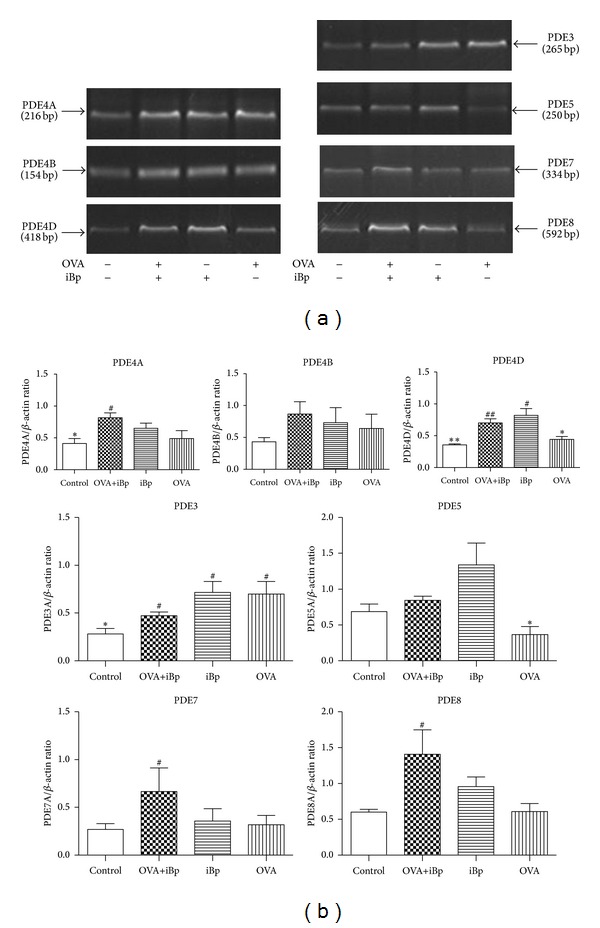
(a) Representative image of PDE subtype mRNA expression. (b) Levels of mRNA expression of lung PDE subtypes in normal (*n* = 8), OVA+iBp, iBp (*n* = 6), and OVA rats (*n* = 8). Data are expressed relative to *β*-actin (*P < 0.05; **P < 0.01 versus OVA+iBp; ^#^ *P* < 0.05; ^##^ *P* < 0.01 versus normal).

To investigate whether the elevation of PDE activity was due to enhanced PDE subtype gene expression, we measured the lung mRNA levels of subtypes PDE1 to PDE11 in control, OVA, OVA+iBp, and iBp rats. There were no significant changes in the expression of PDEs 1, 2, 9, 10, and 11 (data not shown). The expression of PDEs 3, 4A, 4D, 5, 7, and 8 was upregulated in OVA+iBp rats. Although PDE4A and PDE4D were significantly increased in the OVA+iBp rats, PDE4A did not increase in the OVA or iBprats, while simultaneous exposure to OVA and intramuscular iBpenhanced the PDE4A expression. Interestingly, the expression of PDE4D was significantly increased in iBprats, while in the OVA group it did not increase. This suggested that iBpspecially upregulated PDE4D (Figure 5). At the same time, this result also suggested that the increased cAMP-PDE activity might be due to enhanced PDE7 and PDE8 gene expression by simultaneous exposure to OVA and iBp, at least in part.

The expression of PDE3 and PDE5 was complex. In OVA group, PDE5 was downregulated. Interestingly, in iBp rats, PDE5 was significantly increased, while in OVA+iBp group, the level of PDE5 expression was located in the middle of the OVA group and OVA+iBp group. PDE3 was upregulated in OVA or iBp groups, while in OVA+iBp group PDE3 was lower than OVA or iBp groups. This suggested that iBp specially upregulated PDE5, there was not siginificant difference.

### 3.5. Simultaneous Exposure to OVA and Intramuscular iBp Enhanced the Infiltration of Eosinophils

**Figure 6:**
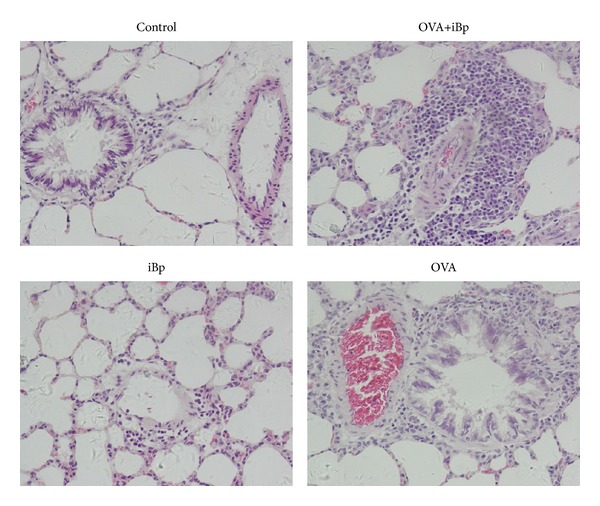
Representative histological alterations of the small airways and small blood vessels in control, OVA+iBp, iBp, and OVA rats.

In the lung histology, increased numbers of infiltrated eosinophils were observed in peribronchial and perivascular tissues in the OVA and OVA+iBp groups, with more eosinophils in the latter. In iBp group, the lung histology was similar to normal controls (Figure 6).

## 4. Discussion

Inactivated* B. pertussis* has long been used as an effective adjuvant for eliciting IgE responses to a variety of antigens in experimental animals, while being also an antigen [16–19]. Because of this specific property, iBp has also been used in animal models of hypersensitivity to boost allergic responses to HDM, OVA, and ragweed pollen. As early as 1980, Bartell and Busse revealed that administration of* B. pertussis* vaccine to mice is associated with the development of impaired *β*-adrenoceptor responsiveness and in many respects resembles human asthma. The relaxant effects of isoprenaline are impaired in tracheal smooth muscle isolated from* B. pertussis-*vaccinated mice [20]. Further, Giembycz suggested that *β*2-adrenoceptor desensitization is based on the accelerated degradation of cAMP by phosphodiesterase [21]. Recently, Xiang et al. not only suggested that PDE4D is an integral component of the *β*2-adrenoceptor signaling complex but also underscored the critical role of subcellular cAMP regulation in the complex control of receptor signaling [22]. So, we assumed that administration of iBp would be associated with the upregulation of PDE4D activity and expression.

Our allergic model described here provided an experimental system that could be used to further investigate the potential role of iBp on the development of allergy and asthma. This study also confirmed the capabilities of iBp. After subsequent pulmonary antigen challenge, presensitized animals displayed many features of allergic asthma including increased bronchial hyperresponsiveness, eosinophilic inflammation, and mucus production. These data suggested that iBp enhances this sensitization process. The AHR induced by iBp has long been known [23, 24], but the mechanism of this effect was unclear. The effects of iBp may be attributed to lipopolysaccharide (LPS) or pertussis toxin. Importantly, both are agonists of Toll-like receptor 4 but have different downstream effects.

In the present study, we investigated PDE regulation in the lung of OVA+iBp, OVA, and iBp rats. OVA sensitization and challenge increased the cAMP-PDE activity which might be primarily due to upregulation of PDE4 and PDE3, but the decreased cGMP-PDE activity might be primarily due to the downregulation of PDE5. There was no significant contribution of iBp to cAMP-PDE activity and cGMP-PDE activity; this result was consistent with the fewer changes in histology and BALF. The main contribution of iBp was to AHR, rather than to inflammation. Our data suggested that* B. pertussis* specifically enhances AHR and specifically enhances the PDE4 activity and PDE4D expression.

Regulation of PDE4 in allergy and asthma has been investigated by many groups but only in human blood leukocytes, and the data are inconsistent. Some groups reported a significant increase in PDE4 activity in asthmatic or allergic patients [25–29], whereas others found no significant difference in the enzyme activity in such patients as compared with healthy individuals [30–33]. PDE4B is involved in the inflammatory process in airways [34]. The only other significant PDE4 gene inducer known thus far is lipopolysaccharide (LPS), which specifically activates the PDE4B gene in monocytes/macrophages [35, 36]. However, in the lungs of allergic rats, expression of the PDE4A and PDE4D genes, but not the PDE4B gene, was upregulated, suggesting that the expression of these genes might be induced by a mechanism(s) distinct from those cAMP or LPS mechanisms. Our result showing upregulation of PDE4 in the lungs of allergic rats supports the use of PDE4 inhibitors in asthma. Thus, if PDE4 does play a role in the pathogenesis of asthma, lung PDE4 may be as important as, if not more important than, leukocyte PDE4. Various PDE4 subtypes are known to be expressed in different tissues and play distinct biological roles. PDE4B is the predominant subtype in blood neutrophils and monocytes [37] but not in lung, which may explain the unchanged PDE4B mRNA expression.

Interestingly, PDE4D gene expression was specially increased in iBp rats. So we assumed there is a relationship between PDE4D and iBp. PDE4A is involved in the inflammatory process in airways [34]. PDE4D gene is involved in emesis [38], which limits the therapeutic use of PDE4 inhibitors. At the same time, PDE4D is also involved in AHR PDE4D-null mice which no longer respond to cholinergic stimulation, and AHR on exposure to antigen is abolished [11]. In addition, animals exposed prenatally but not postnatally to cigarette smoke exhibited increased AHR causally associated with PDE4D mRNA expression in the lung [12]. Recently, PDE4D has been shown to play an important role in vascular diseases, including stroke [39]. These findings demonstrate that the PDE4D gene plays an essential role in the development of some disease. How to benefit from PDE4D and avoid the adverse emesis effect is still an important question.

In summary, our results showed that inactivated* B. pertussis* specifically induces the PDE4D expression and airway hyperresponsiveness, rather than inducing inflammation in the lung and upregulating the total PDE activity.

## Figures and Tables

**Table 1:** Primer sequences of rats PDEs.

Gene	Primer sequences (5′-3′)	Length (bp)	*T*_*m*_ (°C)
*β*-Actin	Sense: AACCCTAAGGCCAACCGTGAAAAG	343	57
	Antisense: GCTCGAAGTCTAGGGCAACATA		

PDE1	Sense: TGGAAGCTGCACTACAGGTG	375	59
	Antisense: CTTCAGGTCCACAGCTGACA		

PDE2	Sense: GAGGACATCGAGATCTTTGC	442	57
	Antisense: TCTTTGTAGATCAGCTCCGC		

PDE3	Sense: TCTTTGCCACTCCTACGACT	265	57
	Antisense: CTGTGCCTGATAAACACTGC		

PDE4A	Sense: TCAACACCAATTCGGAGCTGG	216	61
	Antisense: GTCTTCAGGTCAGCCAGGAGG		

PDE4B	Sense: AGGATTCTGAAGGACCGG	154	56
	Antisense: AGATTATGTGTCGATCAG		

PDE4C	Sense: ACTGAGTCTGCGCAGGATGG	539	62
	Antisense: CACTCCTCTTCCTCTGCTCTCCTC		

PDE4D	Sense: GGCTTCATAGACTACATTG	418	56
	Antisense: TTACACTGTTACGTGTCAGG		

PDE5	Sense: CTGTCTGATCTGGAAACAGC	250	57
	Antisense: GCAATCAGCAATGCAAGCGT		

PDE7	Sense: TCGTATGCTAGGAGATGTCCG	334	55
	Antisense: GCTTACTAGACTATTTCCATTTG		

PDE8	Sense: GGAGAACCAACTCCTTCCTGTG	592	58
	Antisense: AGGCATCCCATGCATCAAAC		

PDE9	Sense: ATGGACCGAGACAAAGTGAC	275	57
	Antisense: AGGCGAACGGTCTTCATTGT		

PDE10	Sense: CTGAGGGGGATGAGATGAAG	322	58
	Antisense: TCAGTTGCTAGGCAGACATCA		

PDE11	Sense: TTCAGCTCGGACAGTCCTAAA	374	58
	Antisense: TCCACTAGCAAAGGAGACGAA		

**Table 2:** Antigen-induced lung inflammatory cells in bronchoalveolar fluid.

Group	*n*	Total number	Eosinophils	Lymphocytes	Neutrophils	Monocytes
		(×10^4^ cells/L)				
Control	6	35.0 ± 12.6	0.12 ± 0.11	31.34 ± 12.31	1.90 ± 0.54	1.64 ± 0.39
OVA+iBp	6	169.6 ± 48.4**	31.73 ± 7.46**	86.13 ± 23.42**	23.45 ± 0.99**	13.20 ± 10.75*
OVA	6	94.0 ± 11.27**	16.54 ± 5.35**	61.90 ± 7.56**	8.93 ± 1.12**	7.38 ± 2.94**
iBp	6	31.0 ± 14.41	1.23 ± 0.73**	22.31 ± 9.21	6.48 ± 3.72*	2.77 ± 1.95

*P < 0.05, **P < 0.01, versus control group.
